# Age-Related Changes in Molecular and Whole Muscle Function: Role of Fat Content?

**DOI:** 10.1093/geroni/igaa057.463

**Published:** 2020-12-16

**Authors:** Joseph Gordon III, Nicholas Remillard, Chad Straight, Rajakumar Nagarajan, Bruce Damon, Stuart Chipkin, Mark Miller, Jane Kent, Joseph Gordon

**Affiliations:** 1 University of Massachusetts Amherst, Amherst, Massachusetts, United States; 2 Vanderbilt University, Nashville, Tennessee, United States

## Abstract

Decreases in muscle size and function are a general consequence of old age; the precise mechanisms of these changes remain unclear. Recent studies suggest that fat deposition in muscle may also contribute to dysfunction in older adults. Fat content was quantified in the quadriceps, and its effects on function in healthy young (21-45 y) and older (65-75 y) men and women (n=44) of comparable physical activity were compared. A subset of the young matched with the older group for muscle fat content were also examined. Peak fat-free whole muscle cross-sectional area (mCSA; cm2), volume (MV; cm3), fat content (fat fraction, FF; %), specific torque (Nm/mCSA) and peak contraction velocity (Nm∙s-1) were determined using fat-water magnetic resonance imaging and dynamometry (0-300□∙s-1). To examine potential molecular mechanisms of muscle weakness, vastus lateralis biopsies were obtained (n=31) and cross-bridge kinetics of type I and II fibers were determined. FF was higher in older adults than young (8.4±1.2% (SE), 7.6±1.4; p=0.03), while mCSA (48.9±10.4 vs. 64.2±17.3), MV (1536±532 vs. 2112±708), specific torque (2.6±0.4 vs. 3.2±0.4), and peak voluntary contraction velocity (422±20 vs. 441±23) were lower in older than young (p<0.01). Type II fiber myosin attachment rate was slower and attachment time longer in older muscle (p<0.017), providing a potential mechanism for the slowing of peak contraction velocity with age. Notably, differences at the whole muscle and molecular levels remained for the subset of young and older groups matched for FF, suggesting that fat deposition in muscle does not exacerbate age-related changes in function.

